# Detection of Endogenous Iron Reduction during Hepatocarcinogenesis at Susceptibility-Weighted MR Imaging: Value for Characterization of Hepatocellular Carcinoma and Dysplastic Nodule in Cirrhotic Liver

**DOI:** 10.1371/journal.pone.0142882

**Published:** 2015-11-25

**Authors:** Ruo-kun Li, Suzanne L. Palmer, Meng-su Zeng, Jin-wei Qiang, Frank Chen, Sheng-xiang Rao, Ling-li Chen, Yong-ming Dai

**Affiliations:** 1 Department of Diagnostic Radiology, Zhongshan Hospital, Fudan University, Shanghai, China; 2 Department of Radiology, Jinshan Hospital, Fudan University, Shanghai, China; 3 Department of Radiology, Keck Medical Center, University of Southern California, Los Angeles, California, United States of America; 4 Department of Pathology, Zhongshan Hospital, Fudan University, Shanghai, China; 5 Siemens Ltd, China Healthcare Sector MR Business, Shanghai, China; Kanazawa University, JAPAN

## Abstract

**Objective:**

To investigate the value of susceptibility-weighted imaging (SWI) for characterization of hepatocellular carcinoma (HCC) and dysplastic nodule (DN).

**Materials and Methods:**

Sixty-eight cirrhotic patients with 89 hepatocellular nodules underwent SWI. The radiological features of hepatocellular nodules on SWI were classified into three types: type A (iso- or hypointensity, and background liver siderosis), type B (hyperintensity, and background liver siderosis), or type C (hyperintensity, and no background liver siderosis). Intranodular and background liver iron content was quantified and correlated with SWI pattern. Prussian blue staining was performed to quantify intranodular and background liver iron content.

**Results:**

Type A pattern (n = 12) contained 11 (91.7%) DNs and 1 (8.3%) HCC, Type B pattern (n = 66) comprised 1 (1.5%) DN and 65 (98.5%) HCCs (including 12 DN-HCCs and 53 overt HCCs), and type C pattern (n = 11) was exclusively seen in HCCs. The iron scores of DN-HCCs and overt HCCs were significantly lower than those of background livers [(0.091±0.30) *VS* (2.18±0.87), P = 0.000; (0.11±0.41) *VS* (2.16±0.97), P = 0.000; respectively]. There was no significant difference between iron scores of DNs and those of background livers [(1.92±0.29) *VS* (2.17±039), P = 0.191]. For lesion-based and patient-based analysis of HCCs (DN-HCCs and overt HCCs), type B pattern showed a sensitivity, specificity, accuracy, positive predicative value (PPV), and negative predicative value (NPV) of 84.4% and 84.4%, 91.7% and 75%, 85.4% and 83.8%, 98.5% and 98.2%, 47.8% and 23.1%, respectively.

**Conclusion:**

SWI can provide valuable information for characterization of HCC and DN based on endogenous iron reduction during hepatocarcinogenesis.

## Introduction

The development of hepatocellular carcinoma (HCC) in cirrhotic liver is usually described as a process of hepatocarcinogenesis, from dysplastic nodule (DN), to DN with microscopic foci of HCC (DN-HCC), and finally to overt HCC [1–4]. Histologically, these nodules demonstrate a series of pathophysiological changes during hepatocarcinogenesis, including vascularity, metabolism, cell number and function, which form the basis of radiological differentiation of HCC and DN at multimodality MR imaging including dynamic gadolinium-enhancement imaging, gadoxetic acid-enhanced imaging, diffusion weighted imaging, et al [5–11].

Iron overload is another common pathophysiological change in cirrhotic liver with prevalence of approximately 22%-67% in nonbiliary cirrhosis and 7%-20% in biliary cirrhosis [12]. Excessive iron accumulation may be a causative factor of chronic liver disease progression and hepatocarcinogenesis [13,14]. Interestingly, HCCs arising in siderotic liver tend to be iron-deficient at pathological analysis, and iron-free foci in siderotic nodules are considered as an early feature during hepatocarcinogenesis [15–21].

Iron usually exists in the form of ferritin and hemosiderin, which can exert superparamagnetic effects and decrease the liver parenchymal signal intensity due to shortening T2 relaxation values on magnet field. T2*-weighted imaging (T2*WI), by gradient echo sequence with long echo time and low flip angle, is usually recommended to detect liver iron deposition with a reported 80% sensitivity and 90% specificity [22,23]. Susceptibility-weighted imaging (SWI) combines T2*WI with the addition of the processed phase information to enhance susceptibility effects caused iron, which has been used for detection and quantification of brain tissue iron [24,25]. Abdominal SWI has been developed for liver imaging which is very sensitive to iron-containing materials. Several studies demonstrated that SWI could improve detection and conspicuity of siderotic nodules in cirrhotic liver and intratumoral hemorrhages within HCCs compared to conventional sequences [26–30]. In the present study, we aimed to investigate the value of SWI for characterization of HCC and DN based on endogenous iron changes during hepatocarcinogenesis.

## Materials and Methods

### Study population

The ethics committee of Zhongshan hospital of Fudan University approved this study and informed consent was waived. One hundred and fifteen consecutive patients undergoing liver mass resection or liver transplantation at our institution from March 2010 to August 2012 were eligible for inclusion in the study. Forty-seven patients were excluded for the following reasons: preoperative regional therapy (n = 5), no cirrhosis confirmed by pathology (n = 18), and non hepatocellular lesions (n = 24) including cholangiocarcinoma, focal nodular hyperplasia, metastasis, angiomyolipoma and hepatoblastoma. The final population included 68 patients with 89 hepatocellular nodules. They were 61 men and 7 women (age range, 22–79 years; mean age 52 years). Sixty-three patients underwent partial hepatic resection, and five patients underwent liver transplantation. The hepatocellular nodules comprised 12 DNs (1.5±0.7 cm), 13 DN-HCCs (2.6±0.7 cm), and 64 overt HCCs (3.9±1.1 cm). Sixty-seven patients had evidence of hepatic viral infection including hepatitis B (n = 63), hepatitis C (n = 3), and both hepatitis B and C (n = 1). The mean interval between MR examination and surgery was 5.8±4.2 days (range, 0–32 days).

### MR imaging

MR imaging was performed with a 3.0-T superconducting magnet (Verio; Siemens, Erlangen, Germany) and a 12-channel body coil. All subjects underwent a routine liver protocol: a respiratory-triggered fat-saturated T2-weighted turbo spin echo (TSE) sequence, breath-hold T1-weighted fast low-angle shot (FLASH) sequence, 3D fat-saturated volumetric interpolated breathhold examination (VIBE) sequence, and dynamic gadolinium-enhancement imaging.

A breath-hold 2D abdominal SWI sequence was performed precontrast with slice thickness, position and number similar to T2-weighted imaging (T2WI). The whole liver was imaged by using three separate breath holds with approximately 10 seconds of free breathing during the whole procedure. Parallel imaging was performed using the generalized autocalibrating partially parallel acquisition (GRAPPPA) with an acceleration factor of 2. SWI post-processing was the same to that used in a previous study [26]. Once SWI was performed, a group of magnitude, phase, minimum intensity projection (minIP), and SWI images were reconstructed online. The parameters of each pulse sequence are summarized in Table 1.

**Table 1:** MR imaging sequences and parameters.

Parameter	TR/TE (msec)	Section thickness (mm)	Intersection gap (mm)	Matrix size	Bandwidth (Hz/pixel)	Flip angle (degree)
T2 TSE	4000/ 78	5	1	168×320	240	140
T1 FLASH	140/ 2.5	5	1	180×320	270	70
T1 VIBE	4/1.4	3	-	180×320	400	9
SWI	150/10	5	1	187×384	189	20

### Image interpretation

Two gastrointestinal radiologists (R.K.L and M.S.Z, with 9 and 24 years of experiences in liver imaging, respectively) who were unaware of histopathological results reviewed MR images in consensus on a commercially available workstation (Syngo Multimodality Workplace; Siemens Healthcare). Firstly, the reader reviewed SWI images to determine the presence of background liver siderosis. Liver siderosis was divided into two patterns: diffuse parenchymal iron deposition and siderotic nodules. Diffuse parenchymal iron deposition appeared as decreased signal intensity of the whole liver compared to that of skeletal muscles. Siderotic nodules appeared as scattered, focal, round, well-demarcated, hypointense foci with superimposed normal signal intensity liver parenchyma [18,26]. Intratumoral hemorrhages were defined as linear, dotlike or patchy hypointensity on SWI, which had been confirmed by SW imaging and pathology correlation in a prior study [29]. Then, conventional MR images were used to confirm hepatocellular nodules (DN, DN-HCC, and overt HCC) and excluded other lesions (cysts and hemangiomas). Finally, the SWI features of hepatocellular nodules were analyzed. The imaging features of hepatocellular nodules on SWI were classified into three types: type A, iso- or hypointense relative to background liver siderosis; Type B, hyperintense relative to background liver siderosis; and type C, hyperintense and no background liver siderosis. The image interpretation results were passed to the study coordinator (S.X.R) for assessment of the correlation of radiological patterns with histopathological results.

### Histopathologic analysis

The hepatocellular nodules and adjacent liver tissue were sampled by a pathologist (L.L.C, with 10 years of experiences in liver pathology) and the study coordinator (S.X.R) to determine the correlation between imaging and histopathology. The pathologist was blinded to clinical status, laboratory results, and imaging information. Double sequential 4-mm-thick paraffin-embedded tissue slices were prepared for hematoxylineosin and Prussian blue staining. Pathological diagnosis of DN and HCC was based on the WHO classification and international criteria for the classification of small hepatocellular nodules. For indeterminate nodules according to morphological criteria, glypican-3 (GPC-3) and glutamine synthetase (GS) immunostaining was further performed to make definite diagnosis [31,32].

Prussian blue staining was performed for identification and quantification of intranodulear and background liver siderosis as follows: 0, none; 1, minimal; 2, mild; 3, moderate; and 4, severe [33]. For the case with a heterogeneous distribution of iron, the highest score was rated to represent its iron score.

### Statistical analysis

The statistical analysis was performed with the SPSS software, Version 16.0. The histopathological iron scores of DNs were compared with those of HCCs using an independent sample t test. The iron scores of DNs and HCCs were compared with those of background livers using a paired-t test. The sensitivity of SWI for detecting background liver siderosis was compared with that of T2*WI using a McNemar test. A *p* value of 0.05 or less was considered to indicate a significant difference.

## Results

### Histopathological iron content of hepatocellular nodule and background liver

Sixty-three (92.6%) of 68 patients had background liver siderosis at pathology, with 17 (27.0%) scored as 1, 22 (34.9%) scored as 2, 18 (28.6%) scored as 3, and 6 (9.5%) scored as 4.

All 12 DNs demonstrated positive iron staining scored from 2 to 4.

One DN-HCC (7.7%) showed iron deposition scored as 1 in HCC foci and scored as 3 in the DN tissue. One DN-HCC (7.7%) had no iron deposition in either HCC foci or DN tissue. The remaining 11 (84.6%) DN-HCCs had no iron deposition in HCC foci and positive iron scored as 2–4 in DN tissue.

Sixty-one (95.3%) of 64 overt HCCs revealed no iron deposition. Three HCCs (4.7%) had iron deposition, one (well-differentiated) scored as 2 and 2 (moderately-differentiated) scored as 1. The comparison of iron scores between hepatocellular nodules and background livers are listed in Table 2.

**Table 2:** Comparison of iron scores between hepatocellular nodules and background livers.

Histological types	hepatocellular nodules	Background livers	P value
DNs (n = 12)	1.92±0.29	2.17±0.39	0.191
DN-HCCs (n = 13)	0.091±0.30*	2.18±0.87	0.000
Overt HCCs (n = 64)	0.11±0.41	2.16±0.97	0.000
≤2cm (n = 25)	0.22±0.65	1.89±0.90	0.000
>2cm (n = 39)	0.053±0.23	2.29±0.98	0.000

* mean iron score of HCC foci

### Radiological patterns of hepatocellular nodules on SWI and histopathological correlation

The radiological patterns of hepatocellular nodules on SWI are summarized in Table 3.

**Table 3:** Radiological patterns of hepatocellular nodules on SWI.

Histological types	SWI pattern		
	Type A	Type B	Type C
Dysplastic nodule (DN)	11 (91.7%)	1 (1.5%)	-
DN-HCC	-	12 (18.2%)	1 (9.1%)
Overt HCC	1 (8.3%)	53 (80.3%)	10 (90.9%)

**Fig 1:**
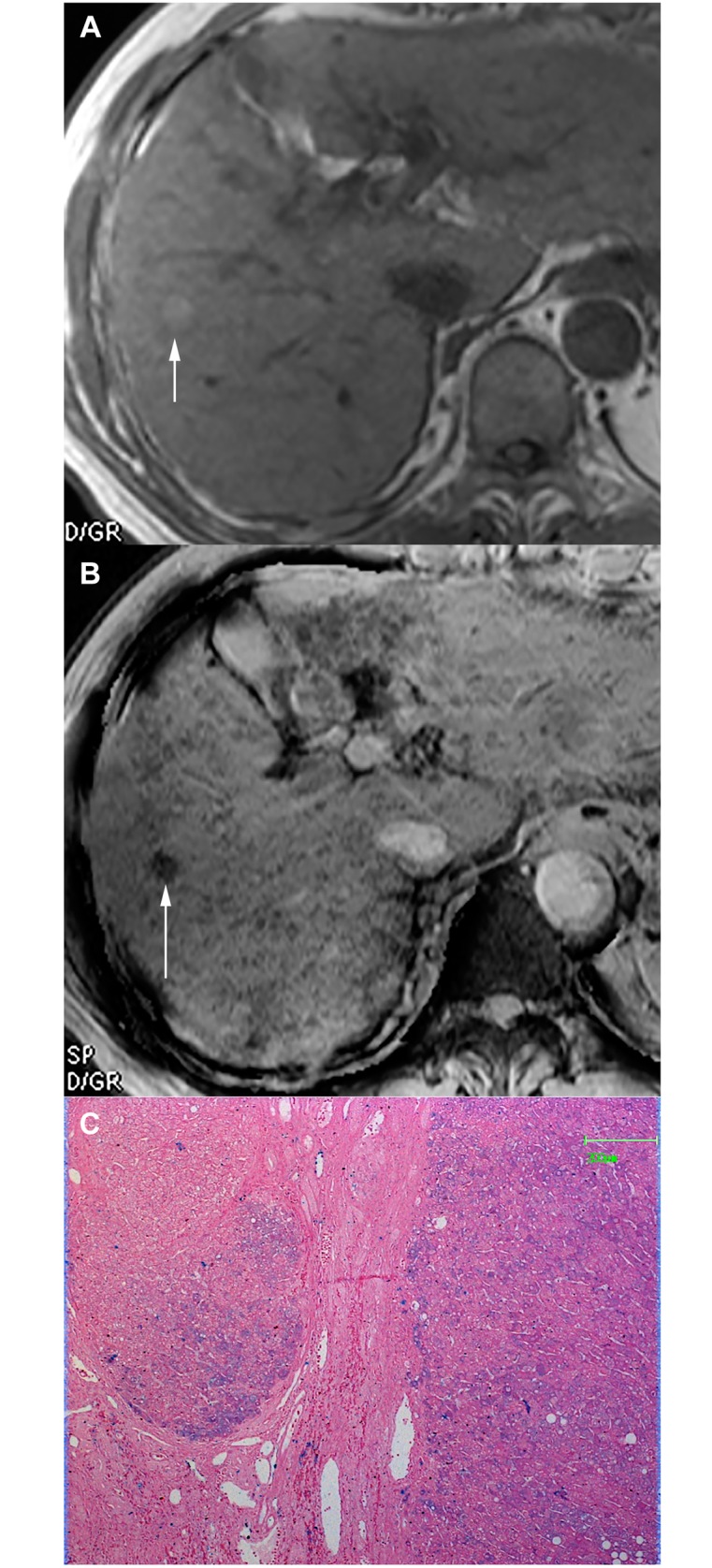
DN appearing as Type A pattern. (a) Transverse T1–weighted image demonstrates a hyperintense nodule in segment V(arrow). (b) The nodule appeared as focal homogenous hypointensity (arrow) with diffuse siderotic nodules in background liver. (c) Photomicrography of Prussian blue staining slide demonstrates iron deposition scored as 3 in the nodule (×50). The patient also had another HCC lesion measuring 1.9cm (not shown slice) who undergone liver transplantation.

**Fig 2:**
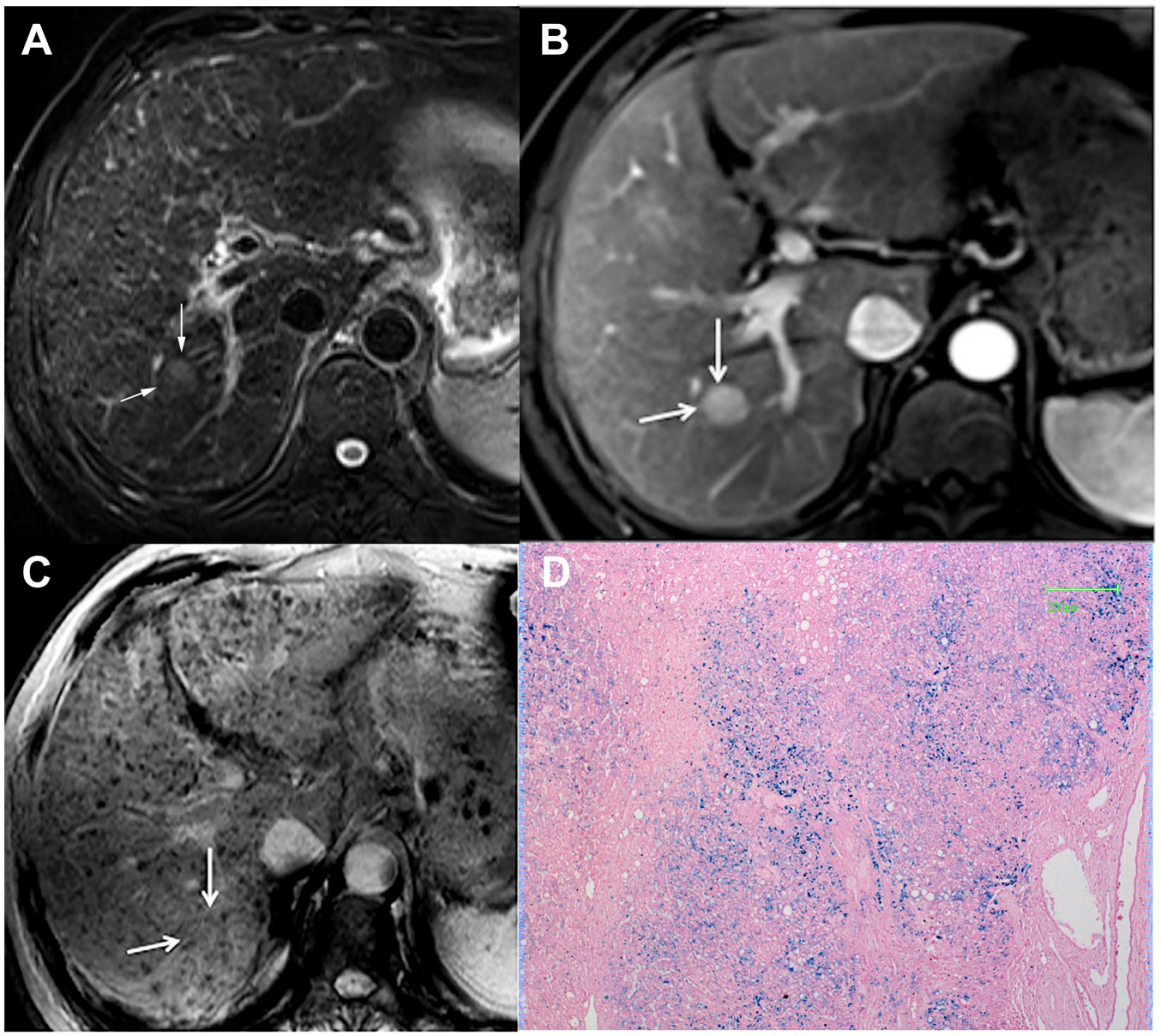
DN appearing as Type A pattern. (a) Transverse T2–weighted image demonstrates a slightly hyperintense nodule in segment VI (arrow). (b) Transverse arterial phase image demonstrates an intense enhancing nodule (arrow). (c) On SWI, the nodule appeared as isointensity with internal tiny siderotic nodules similar to background liver. (d) Photomicrography of Prussian blue staining slide demonstrates iron deposition scored as 4 in the nodule (×50). The nodule was preoperatively misdiagnosed as HCC.

Type A pattern (n = 12) contained 11 (91.7%) DNs and 1 (8.3%) HCC. Nine DNs appeared as round, well-demarcated, homogeneous hypointensity (Fig 1). Two DNs and 1 HCC demonstrated isointensity with multiple internal tiny siderotic nodules similar to background liver siderosis (Fig 2).

**Fig 3:**
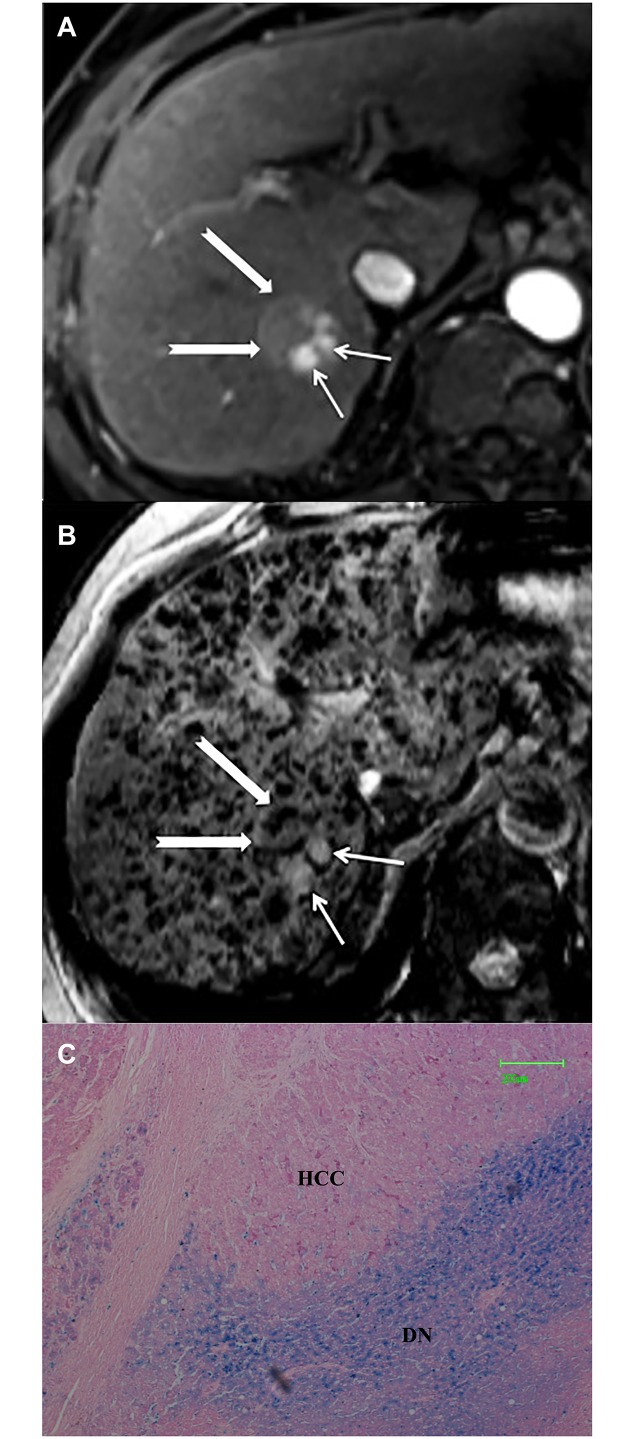
DN-HCC appearing as Type B pattern. (a) Transverse arterial phase image demonstrates an intense enhancing inner nodule (arrow) and no enhancing outer nodule in segment VIII (open arrow). (b) On SWI, the inner nodule appeared as hyperintensity (arrow) and outer nodule appeared as hypointensity (open arrow) with multiple siderotic nodules in background liver. The nodule was chracterized as type B pattern (nodule-in-nodule). (c) The nodule was confirmed as DN-HCC by surgery pathology. Photomicrography of Prussian blue staining slide demonstrates iron-free HCC foci and siderotic noncancerous DN tissue scored as 4 (×50).

Type B pattern (n = 66) comprised 1 (1.5%) DN and 65 (98.5%) HCCs (including DN-HCCs and overt HCCs). One DN was 2.8 cm in size and demonstrated heterogeneous intranodular iron deposition scored from 1 to 3, with background liver siderosis scored as 3 (Fig 3). HCCs generally appeared as nodular iron-free areas relative to background liver siderosis (scored as 2–4). Internal signal characteristics on SWI and corresponding histopathology were described as follows in detail:

*—DN-HCCs*. (1) Hyperintense inner nodules within hypointense outer nodules (nodule-in-nodule) (n = 4). Pathologically, outer nodules had iron deposition the same as background liver in 3 lesions and slightly lower than background liver in 1 lesion; one inner nodule had iron deposition scored as 1, and 3 inner nodules had no iron deposition (Fig 3). (2) Homogeneous hyperintensity relative to background liver siderosis (n = 7). HCC foci in 6 lesions had no iron deposition and HCC foci in one lesion was scored as 2. (3) Mosaic hyperintensity (n = 1), consistent with internal hemorrhage at histopathology. No iron deposition were identified in viable tumor components.

**Fig 4:**
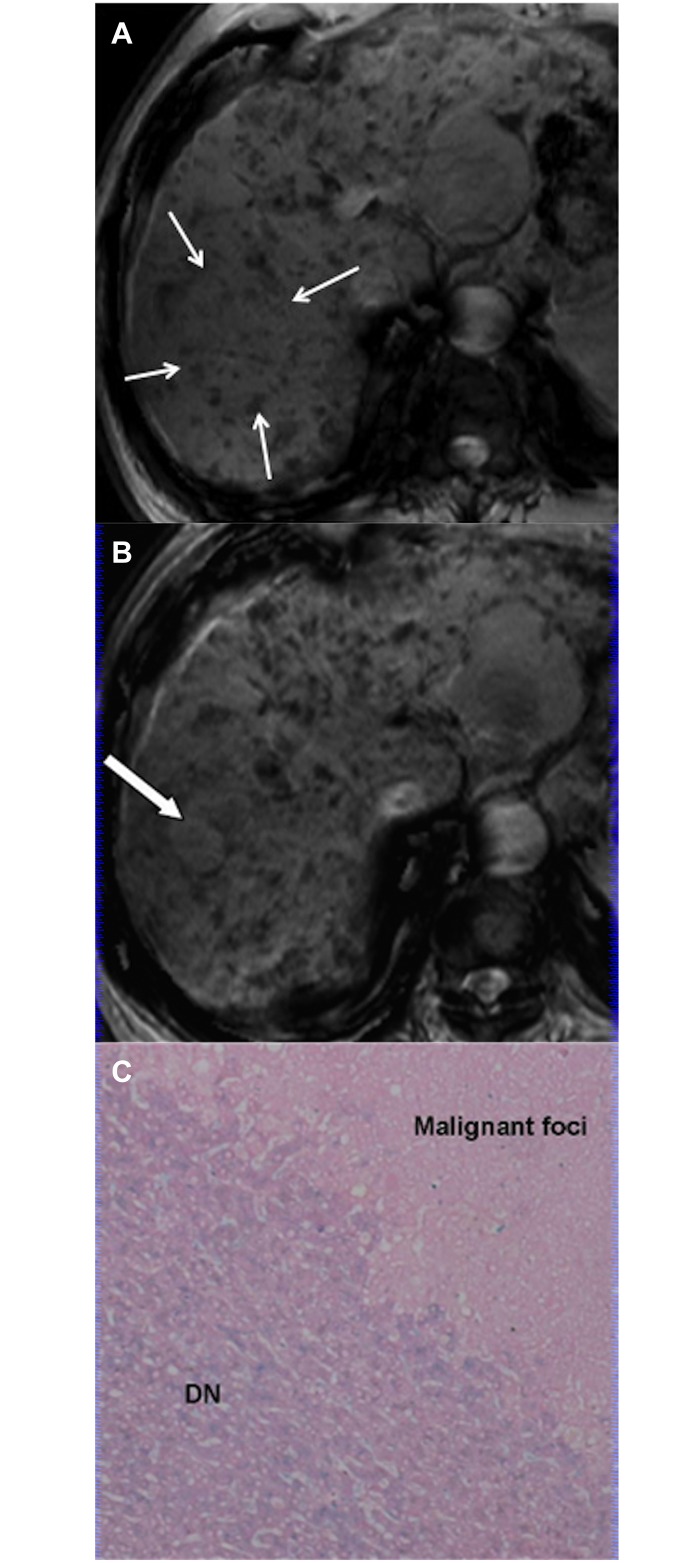
DN-HCC. (a) Prior SWI demonstrates an isointensity nodule with internal tiny siderotic nodules similar to background liver, consistent with type A pattern (arrow). (b) Follow-up SWI after 14 months interval demonstrated a hyperintensity inner nodule, consistent with type B pattern (arrow). (c) The nodule was confirmed as DN-HCC by surgery pathology. Photomicrography of Prussian blue staining slide demonstrates iron-free HCC foci and siderotic noncancerous DN tissue scored as 3 (×50).

In addition, one patient had twice SWI images. The prior SWI image showed the nodule as type A pattern. However, the follow-up SWI image after 14 months interval demonstrated that it evolved into type B with a nodule-in-nodule hyperintensity. Consequently, it was confirmed as a DN-HCC by surgery pathology. No iron deposits was identified in the HCC foci, but iron deposits of scoring 3 was demonstrated in the outer nodule and background liver (Fig 4).

**Fig 5:**
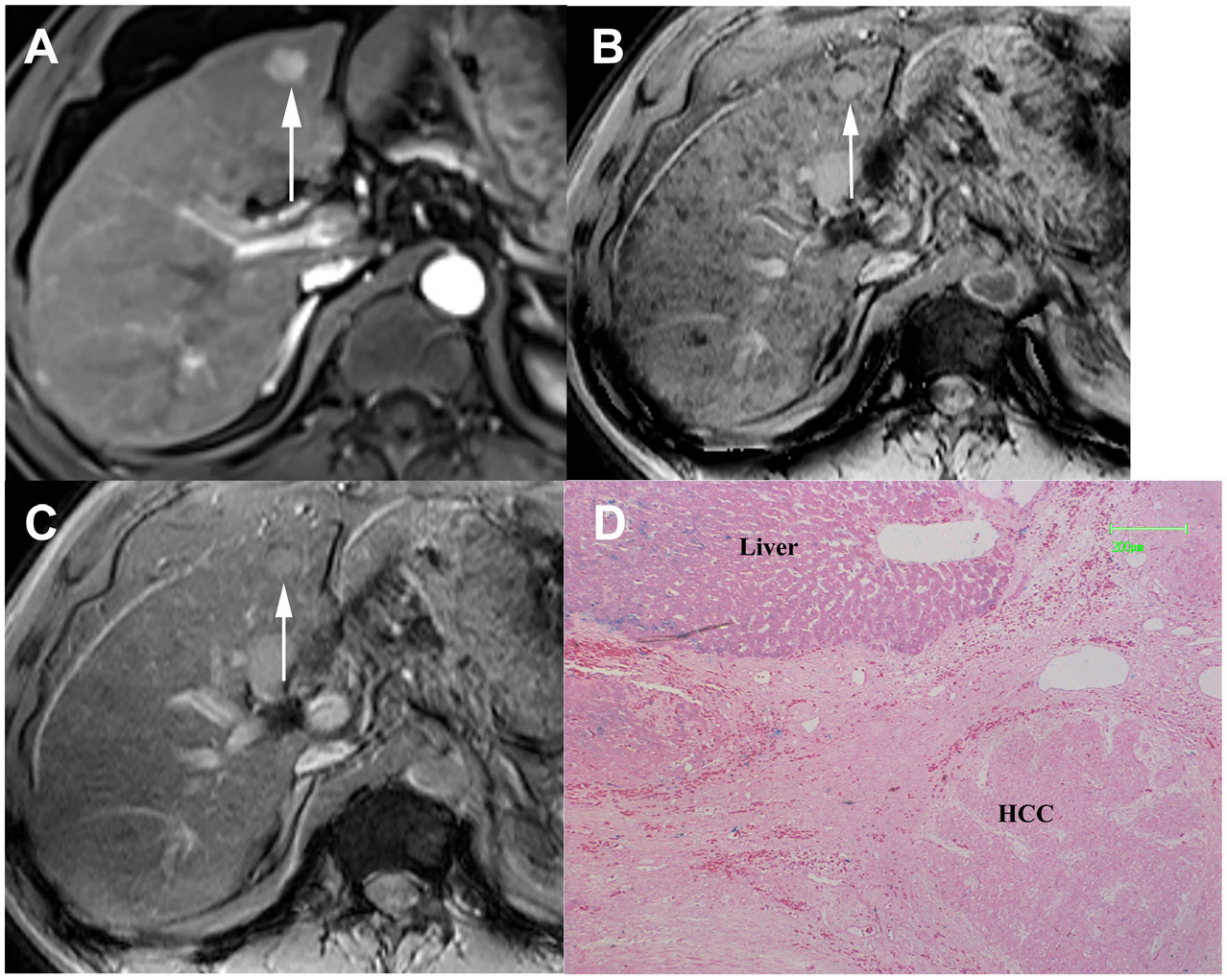
HCC appearing as Type B pattern. (a) Transverse T1WI arterial phase image demonstrates an intense enhancing nodule in segment in segment IV (arrow). (b) On SWI, the nodule appeared as homogenous hyperintensity with multiple siderotic nodules in background liver (arrow). (c) The nodule was characterized as type C pattern on T2*WI due to lack of detection of background liver siderosis (arrow). (d) Photomicrography of Prussian blue staining slide demonstrates no iron deposition in the nodule and iron deposition scored as 2 in the background liver (×50).

**Fig 6:**
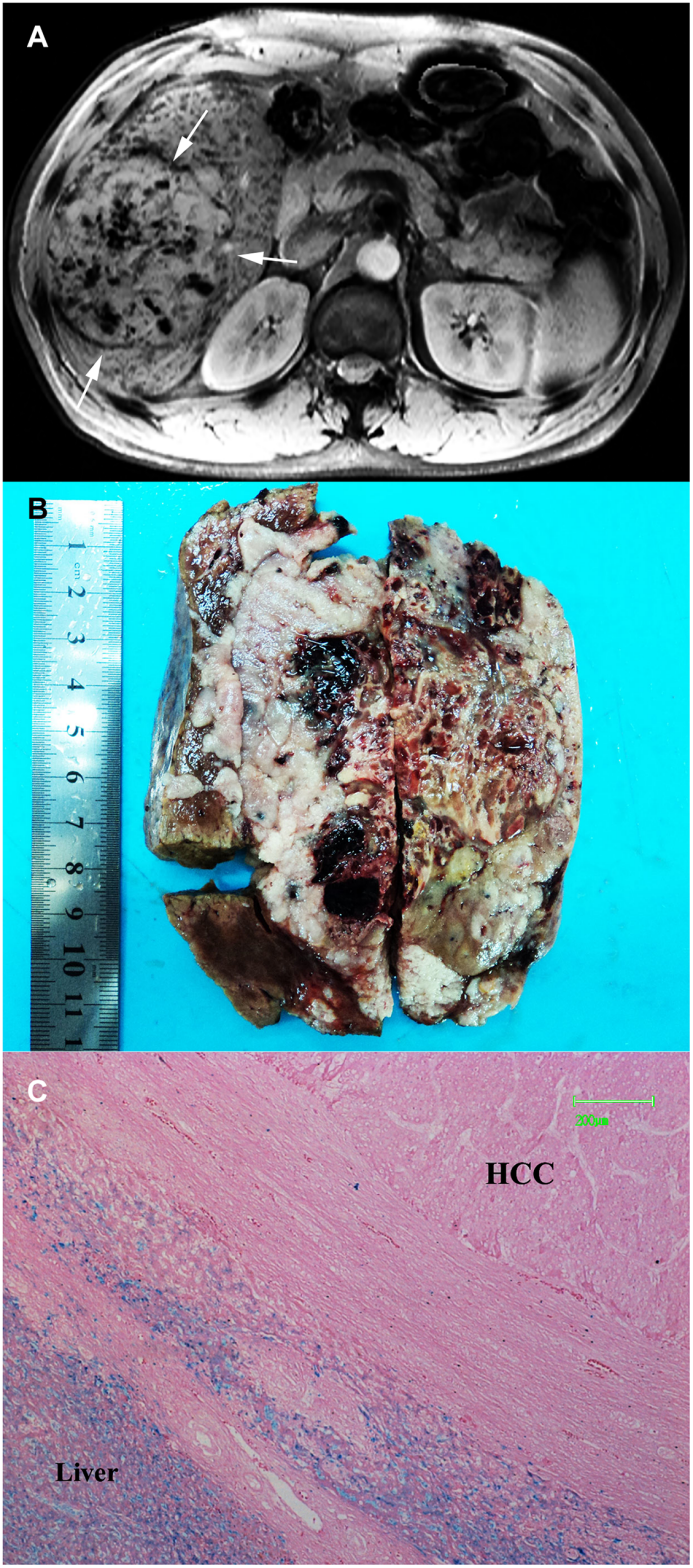
HCC appearing as Type B pattern. (a) SWI demonstrates a mosaic hyperintensity mass with multiple siderotic nodules in background liver in segment V and VI (arrow). (b) Grossly surgical specimen reveals patchy intratumoral hemorrhages. (c) Photomicrography of Prussian blue staining slide demonstrates no iron deposition in the vial component of HCC and iron deposition scored as 4 in the background liver (×50).

*—Overt HCCs*. (1) Homogeneous hyperintensity (n = 35), consistent with relative or absolute lack of intranodular iron deposition compared to background liver (Fig 5). As mentioned above, 3 HCCs had iron deposition of scored as 2, 1, and 1 associated with background iron deposition scored as 2. (2) Mosaic hyperintensity (n = 18), consistent with intratumoral hemorrhage, but no iron staining in viable tumor components at pathology (Fig 6).

**Fig 7:**
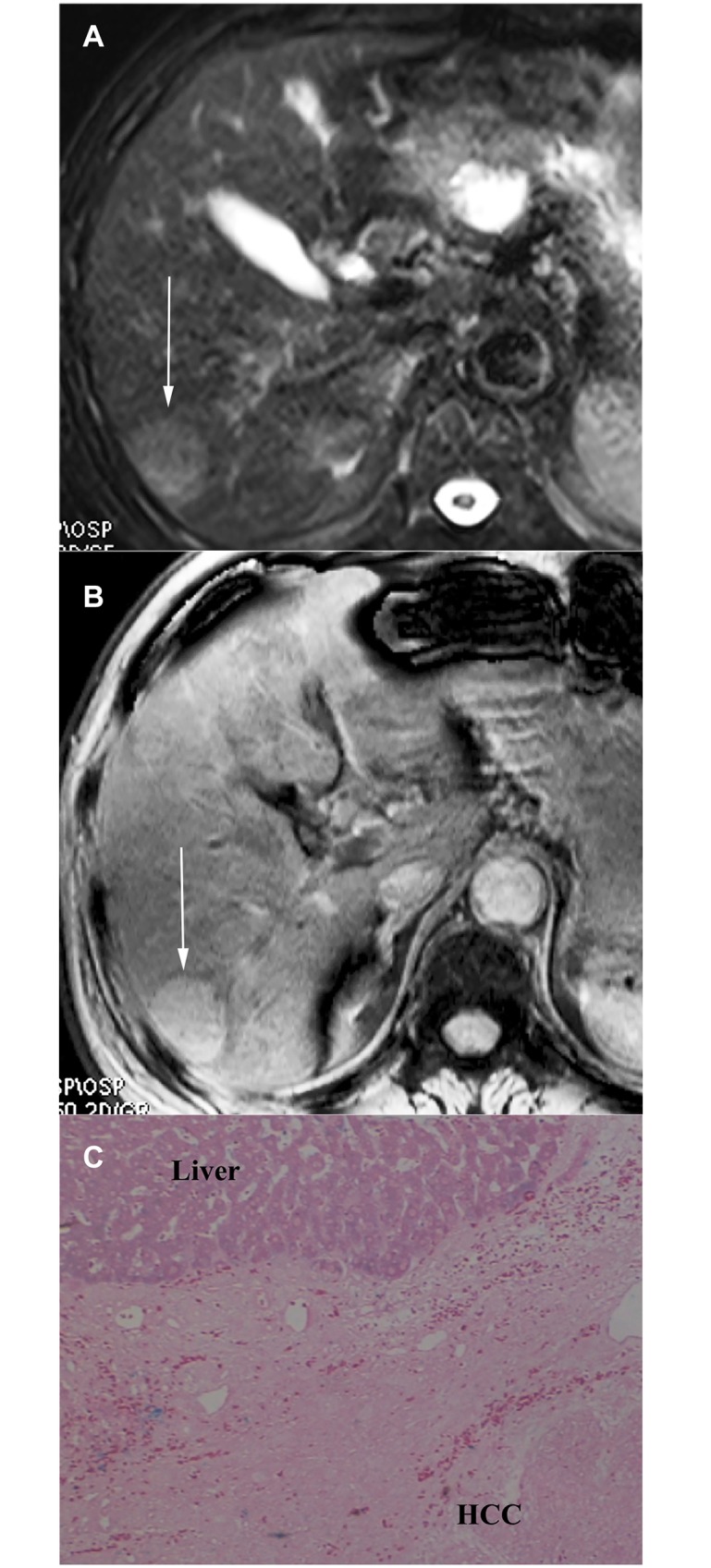
HCC appearing as Type C pattern. (a) Transverse T2–weighted image demonstrates hyperintense mass in segmentVI (arrow). (b) On SWI, the mass appears as hyperintensity without background liver siderosis (arrow). (c) Photomicrography of Prussian blue staining slide demonstrates no iron deposition in either HCC or background liver (×50).

Type C pattern (n = 11) was exclusively seen in HCCs. In this group, 1 patient had 1 DN-HCC and 1 overt HCC, and another patient had 2 overt HCCs. On SWI, 3 overt HCCs appeared as mosaic hyperintensity consistent with intratumoral hemorrhage at pathology. The remaining 8 lesions appeared as homogeneous hyperintensity. No iron deposition was demonstrated in these 11 lesions (Fig 7). However, 3 patients had iron deposition scored as 1 and 1 patient had iron deposition scored as 2 on background liver. The remaining 5 patients had no background liver iron deposition.

### Differentiation of HCC from DN by radiological pattern on SWI

For lesion-based analysis of HCC, type B pattern had a sensitivity, specificity, accuracy, positive predicative value (PPV), and negative predicative value (NPV) of 84.4%, 91.7%, 85.4%, 98.5%, and 47.8%, respectively. For patient-based analysis, it showed a sensitivity, specificity, accuracy, PPV, and NPV of 84.4%, 75%, 83.8%, 98.2%, and 23.1%, respectively.

**Fig 8:**
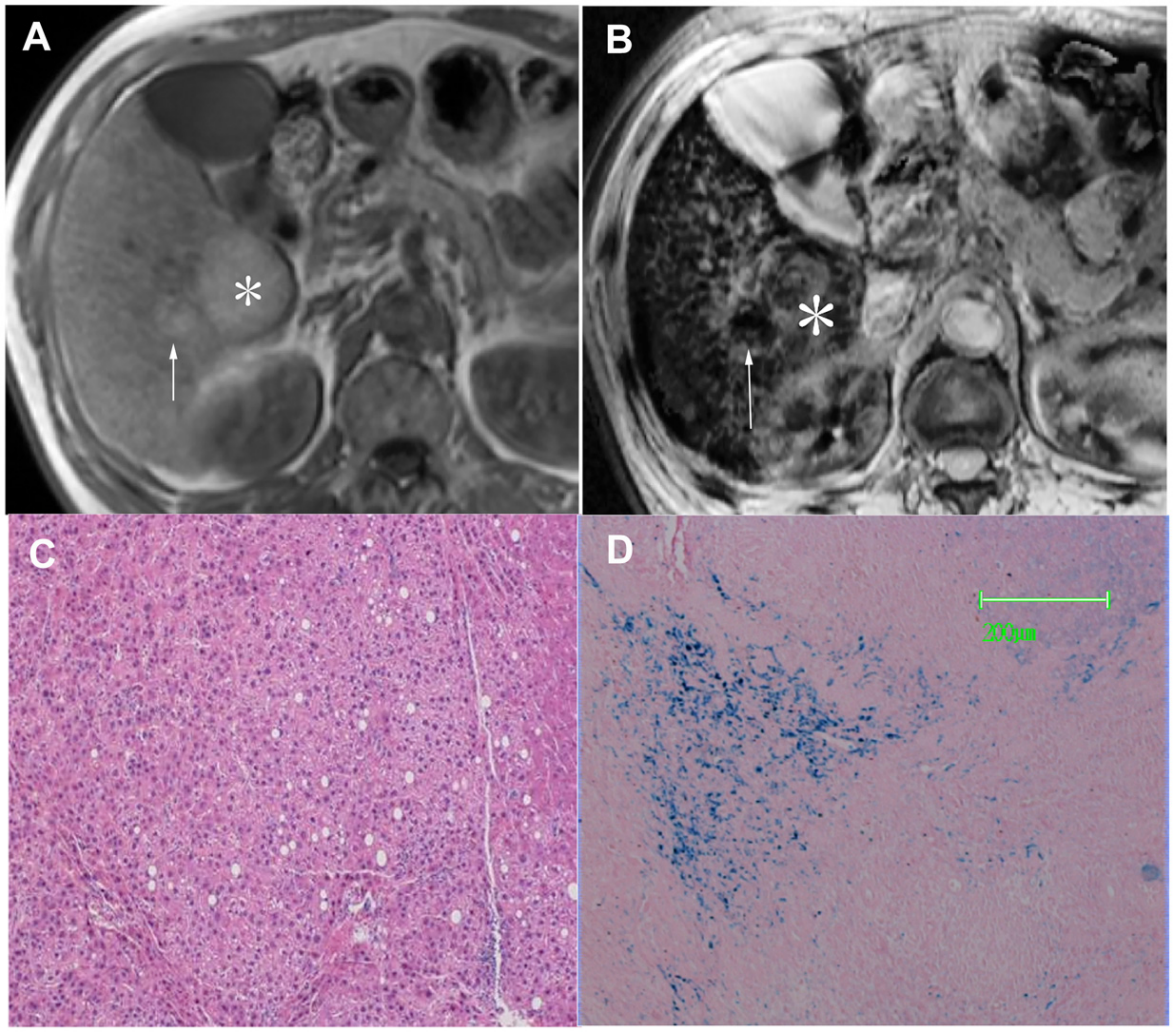
DN appearing as Type A pattern. (a) Transverse T1-weighted image demonstrates a hyperintense nodule in segment VI measured 2.8 cm (asterisk). A adjacent smaller hyperintense nodule is present (arrow). (b) The nodule appeared as heterogeneous hyperintensity with background liver siderosis on SWI, consistent with type B pattern (nodule-in-nodule) (asterisk). The adjacent smaller nodule appeared as homogeneous hpypointensity consistent with type A pattern (arrow). (c) Photomicrography of HE staining slide confirms the diagnosis of DN with active proliferation. The adjacent smaller nodule is also confirmed as a DN (not shown). (d) Photomicrography of Prussian blue staining slide demonstrates heterogeneous iron deposition in the nodule scored as 1–4 (×50).

*—False positive*. One DN was characterized as type B pattern on SWI. At pathology, the nodule showed active proliferation and heterogeneous iron staining from grade 1 to 3 (Fig 8).

*—False negative*. One HCC was characterized as type A pattern due to similar intranodular iron deposition appearing similar to background liver. Four HCCs were characterized as type C pattern because background liver siderosis was not identified (scoring 1 in 2, and scoring 2 in 1).

### Comparison for characterization of radiological pattern between SWI and T2*WI

Comparison of the sensitivity for detection of background liver siderosis between SWI and T2*WI was summarized in Table 4. For 63 patients with positive background liver siderosis, the overall sensitivity of SWI was significantly higher than that of T2*WI (93.6% vs 63.5%, p = 0.002), attributing to improved detection of iron deposition scored as 1 (76.5% vs 23.5%, p = 0.027)and 2 (100% vs 54.5%, p = 0.037). Background liver siderosis scored as 3 and 4 could be detected by both SWI and T2*WI.

**Table 4:** Comparison of the sensitivity for detection of background liver siderosis between SWI and T2*WI.

	Score 1 (minimal, n = 17)	Score 2 (mild,n = 22)	Score 3 (moderate, n = 18)	Score 4 (severe, n = 6)	Total (n = 63)
T2*WI	4(23.5%)	12(54.5%)	18(100%)	6(100%)	40(63.5%)
SWI	13(76.5%)	22(100%)	18(100%)	6(100%)	59(93.6%)
P value	0.027	0.037	-	-	0.002

T2*WI missed 19 patients with background liver siderosis compared to SWI. These 19 patients had 21 nodules which appeared as type B pattern on SWI but were characterized as type C pattern on T2*WI (Fig 4c). Of these 19 patients, 1 had three nodules including 1 DN-HCC and 2 HCCs, and 18 more patients each had single HCC. Five HCCs showed homogeneous hyperintensity on T2*WI otherwise mosaic hyperintensity on SWI, consistent with intratumoral microbleeds confirmed by pathology.

## Discussion

Our study demonstrates that DN typically appears as intranodular iron deposition and HCC appears as iron-free foci in siderotic liver. Type B pattern on SWI correlates well with intranodular iron reduction compared to background liver.

Terada et al first described *in vitro* 1.5T MRI T2WI characteristics of hepatocarcinogenesis and correlation with intranodular iron content. In their study, iron-poor malignant foci in iron-accumulating adenomatous hyperplastic nodules were recognized as isointense foci, while noncancerous iron-rich liver tissue were hypointense, suggesting the possibility of characterization of HCC based on endogenous iron changes during multistep hepatocarcinogenesis [19]. However, it is challenging to discriminate iron-free HCC and benign siderotic nodules *in vivo* because iron deposition usually is heterogeneous and mild in cirrhotic liver unrelated to hereditary hemochromatosis. Previous studies demonstrated that SWI could improve detection and conspicuity of siderotic nodules in cirrhotic liver compared to T2*WI because it utilized high-filter processed phase information combined with T2*WI to enhance susceptibility effects caused by iron [26–28]. In our present study, a radiological-pathological correlation demonstrated that SWI significantly improved detection of mild iron deposition compared to T2*WI. SWI identified 19 patients with minimal or mild iron deposition missed by T2*WI. Then, 21 type C pattern nodules on T2*WI were characterized as type B pattern on SWI. These findings suggest that SWI demonstrates better ability of detecting endogenous iron reduction during multistep hepatocarcinogenesis compared to T2*WI.

SWI may aid in the characterization of HCC and DN based on endogenous iron content changes, as supported by the results of our study. SWI showed a sensitivity, accuracy, and PPV of 84.4% and 84.4%, 85.4% and 83.8%, 98.5% and 98.2% for lesion-based and patient-based analysis, respectively. This was consistent with the study by Hardie et al. They found that T2*WI had a sensitivity and specificity of 60% and 100% for identifying HCC and a greater degree of liver iron deposition allowed for increased conspicuity of HCC [34–36]. However, it was not confirmed by iron-staining pathologic correlation. In the present study, we only focused on feasibility of SWI for characterization of HCC and DN based on the difference of intranodular iron content. The purpose of our study was not to evaluate the diagnostic performance of SWI compared with other imaging techniques including DWI, gadoxetic acid-enhanced imaging, which should be further illustrated in the further study.

We have to emphasize the fact that the presence of background liver siderosis is the premise for characterization of HCC by SWI. However, liver siderosis is not always accompanied with HCC development. Type C pattern nodules are indeterminate on SWI, which may be a major factor of relatively low NPV (47.8% for lesion-based and 23.1% for patient-based analysis). Moreover, type B pattern could be encountered in other benign lesions, and combination with conventional sequences is essential for accurate interpretation of SW images.

Our study results were somewhat different from previous reports. As to background liver siderosis, a much higher prevalence (92.6%) was present compared to previous reports [12]. This may attribute to the fact that vast majority of population were HCC patients with viral hepatitis in our study sample. In addition, all DNs demonstrated iron deposition, which was much higher than report by Terada et al (approximately 25%) [20,21]. In our study, most DNs were resected nodules visible on conventional MR sequences. DNs should also be present for patients appearing as type C pattern. Hence, the prevalence of siderotic DN should be greatly overestimated in our study. However, SWI only can aid to detect the iron-free foci in siderotic nodules and background liver siderosis. This selection bias does not alter our study conclusion. Fuethermore, our study demonstrated similar degree of iron deposits between DN and background liver. In the study by Terada et al, siderotic DN may selectively accumulate iron to a greater degree [21]. This may be due to different evaluation method that we generally described RNs and DNs as background liver.

Our study had several limitations. First, Prussian blue stains only hemosiderin and does not reflect the exact iron quantity. Further studies with quantification of tissue iron content are necessary in further study. Second, iron deposition in cirrhotic liver usually varies from site to site. In the present study, due to limitation of surgical sample, we only evaluated peritumoral liver tissue which might be insufficient to represent the whole liver. However, detection of HCC mainly depended on local contrast between the tumor and peritumoral liver tissue. Therefore, we believe that it does not affect our study conclusion. Third, as described above, the prevalence of siderotic DN should be greatly overestimated. A thorough explanted liver specimen correlation with imaging should be necessary. Fourth, we excluded biopsied cases to allow pathologic evaluation of the entire lesion. However, many DN were confirmed by biopsy or imaging follow up in clinical setting. This may lead to another selection bias. Fifth, some patients had multiple HCC lesions, resulting in a potential bias because of the clustering effect. Therefore, we analyzed the diagnostic performance of SWI on the basis of both a lesion-based and a patient-based analysis. Finally, the spatial resolution of SWI was limited. Further study is required to improve image quality with a higher matrix size. We did not do calibration with an independent test standard. In addition, we did not compare the diagnostic value of SWI with other imaging techniques including DWI and DCE-MRI, which should be performed in further study.

In conclusion, our study results demonstrate that SWI can accurately detect endogenous iron reduction during multistep hepatocarcinogenesis, which may provide valuable information for characterization of HCC and DN in cirrhotic liver.
